# Challenges Faced by Parents Raising Children With Intellectual Disability in Selected Village in Limpopo Province

**DOI:** 10.1111/jar.70257

**Published:** 2026-07-21

**Authors:** V. Baloyi, N. Maanda, M. Chueng

**Affiliations:** ^1^ Department of Psychology University of Venda Thohoyandou South Africa; ^2^ Research Office, Faculty of Health Science, University of Venda Thohoyandou South Africa

**Keywords:** child, intellectual disability, parents, psychosocial

## Abstract

**Background and Aim:**

The number of children with intellectual disability has increased and remains a phenomenon that requires attention in research. Hence, the current study sought to explore challenges faced by parents raising children with intellectual disability at Tshisaulu village in Thulamela Municipality.

**Methods:**

A qualitative method using an exploratory design was used to gain an understanding of participants' views and experiences of challenges faced in raising a child with intellectual disability. A total of 10 participants at Tshisaulu village were purposively sampled. Data was collected through interviews and analysed using reflexive analysis.

**Results:**

The study findings revealed that parents face psychosocial isolation, stigma and unacceptance by society.

**Conclusion:**

It was recommended that awareness campaigns about intellectual disability be rendered in the community. Social workers, psychologists or mental health workers could work together with the community to conduct educational campaigns about disability in the community.

## Summary

- Raising a child with intellectual disability can be challenging for parents. In rural areas, these challenges can be even greater. This study looked at the difficulties faced by parents raising children with intellectual disability in a village in Limpopo Province.
- Parents struggle to access basic services like healthcare, education and therapy for their children. Lack of family, community and government support makes it harder for parents to care for their children. Limited resources and information about intellectual disability make it difficult for parents to provide the best possible care.
- This study highlights the need for greater support and resources for parents raising children with intellectual disability in rural areas. Governments, communities and organisations must work together to provide accessible services, education and support to help these families thrive.

## 1 Introduction

Over 100 million individuals worldwide live with disability, which represents 15% of the global population or one out seven, 110 million to 190 million adults have major functional challenges (WHO and World Bank 2011). Approximately 266 million children aged 0–19 years are expected to have moderate to severe disabilities, UNICEFF (2012) estimated prevalence of 10.1%. In contrast, the Global Burden Diseases (2019) estimated that 49.8 million children under the age of five, 241.5 million children aged 5–19 years and 291.3 million children under the age of 20 have moderate to severe disabilities, with intellectual disability (ID) being the most common among children under five.

There were approximately 29 million disabled individuals in Nigeria in 2018 (World Bank 2020). The Nigeria Demographic and Health Survey (2018) shows that 7% of household members over five and 9% of households with people aged 60 and over have some degree of difficulty in at least one functional domain, such as seeing, hearing, communicating, cognition or walking, and 1% either have a lot of difficulty or cannot function at all in at least one domain.

Disability increased from 1.4% for those aged 0–14 to 3.1% for those aged 15–65 (Ghana Statistic Service 2014). Approximately 39% of all Ghanaians with disabilities had various disabilities. The 1990s saw the final collection of reliable South African data on the prevalence of intellectual disabilities in children aged 2–9 (Kromberg et al. 2008). Children with impairments ages 0–4 years were not profiled in the 2011 census, and there was no direct measurement of ID (Statistics South Africa 2014).

Moreover, the most recent valid South African ID data, 3.2% of adults aged five and older have mild memory and attention problems, and 1% have severe memory and concentration problems (SSA 2014). In contrast, 3.6% of the children in eight communities who were screened in their homes for childhood disorders in the northern section of South Africa's Mpumalanga province had an ID (Kromberg et al. 2008). UNICEF (2012) highlighted that there were approximately 10,000 children with an ID and approximately 68,000 children with a severe disability in the province of Limpopo. There was no information on the prevalence rate of ID in Thulamela Municipality, and not much research had been done. Regardless of the lack of recorded data in Thulamela municipality concerning the phenomenon, Limpopo province records a high number of ID cases, which warrants research in Tshisaulu village. Parents are the most dependable and lifelong carers for children with intellectual disabilities, and they have a significant influence on how their children develop and experience life (Mazzuchelli et al. 2006). Parents face numerous and co‐occurring challenges, including the time and financial strain of medical care and other therapeutic interventions, limitations on social activities and adjustments to family objectives and accomplishments. In addition, there are not enough support services available in villages for parents of children with intellectual disabilities, which could help parents manage their stress (Katherene 2021). Hence, the current study seeks to explore challenges faced by parents raising children with intellectual disabilities at Tshisaulu Village in Thulamela Municipality.

## 2 Methodology

### 2.1 Research Approach and Design

The current study used a qualitative research approach. It employed exploratory research designs to gain an in‐depth understanding of the phenomenon; the researchers explored challenges faced by parents raising children with intellectual disabilities. This approach was suitable given the limited existing research and allowed flexibility in exploring emerging themes without predetermined hypotheses.

### 2.2 Population, Sampling and Sample Size

The population of this study was male and female parents raising children living with ID located at Tshisaulu village in Thulamela Municipality, Limpopo Province. Tshisaulu is in a rural area where most people are Tshivenda language speakers who value cultural beliefs. The researcher used purposive sampling to select 10 participants at the Diiteleni day care centre for children living with disabilities in Tshisaulu, where they usually pick up their children. The decision to select participants using purposive sampling was based on the fact that they possessed the characteristics the researchers sought, and only 10 participants agreed to take part in the study. The reason other participants did not provide consent to increase the sample size was due to the emotional nature of the study on discussing the impact of parenting children with disabilities. The researcher requested participation from the participants during weekdays after school at the school premises as they picked up their kids. The researcher was the one who selected participants who could provide the information that was needed based on their judgment.

### 2.3 Data Collection

As part of the permission to conduct a study, the researcher sought permission from all relevant spectra. The researchers scheduled interviews with the participants, and parents were interviewed at their homes. Semi‐structured interviews guide using an interview guide was used for data collection. The interview guide‐out was the most appropriate data collection instrument given the nature of the study, and interviewing participants in their homes helped the researchers make sense of participants' feelings, experiences, social situations and the phenomena in their natural settings. Interviewing participants in their homes provided a comfortable, quiet, familiar environment that encouraged openness and honest responses. Additionally, home‐based interviews were convenient for participants and helped to build rapport and improve participation. The interview guide was structured into two sections: Section A: Demographic information of the participants; and Section B: interview questions. Section B included questions such as ‘What were your initial reactions when you learned about your child's condition?’ ‘What challenges do you face caring for your child?’ and ‘What helps you cope with the challenges you face?’ The researchers translated the data collection instrument from English into Tshivenda, as the language that the participants understood better. The interviews lasted 30–45 min, and the researchers recorded them using an audio recorder and field notes, with participants' consent. Data was analysed using reflexive thematic analysis.

### 2.4 Ethical Considerations

The researcher obtained ethical clearance from the University of Venda Research Ethics Committee prior to data collection (reference no. FHS/23/PHYCH/25/1708). Additionally, the researcher introduced the study's purpose to the day care centre officials prior to data collection. This was done through formal communication, such as a permission letter and/or an introductory meeting, to explain the aims of the study and request access to participants. Written and verbal consent was granted by the day care centre manager. Consent was also obtained from the participants who agreed to be part of the study verbally and through signed consent forms. The study assured participants of confidentiality to maintain the safety and privacy of any information, which were ensured through using mock identities such as naming participants such as ‘Participant 2’ instead of a true name. Following data collection, data sheets and the recording device were stored in a lockable drawer and the recordings were also copied to an encrypted folder in a computer for backup purposes. The researchers conducted a debriefing session after the data collection process. However, the researcher did not identify any emotional or psychological harm. In the case that any potential emotional and psychological harm would have arisen, the researchers would have referred the participants to relevant professionals such as Social Workers or Psychologists at the nearby health facility (Tshilidzini Hospital). Data was kept safe to ensure confidentiality, and real names were not used to ensure the anonymity of participants.

### 2.5 Data Analysis

The researcher analysed data using Thematic Content Analysis. The researcher employed thematic data analysis, which is a method for systematically identifying and organising, offering insight into patterns of meaning across a data set. Thematic data analysis allowed the researchers to see and make sense of collective meaning and experiences, and various themes and subthemes were generated during the analysis, which increased the understanding of the phenomenon.

### 2.6 Trustworthiness of the Study

Four criteria for developing trustworthiness were used: namely, credibility, dependability, conformability and transferability. Credibility was ensured through prolonged engagement, reflexivity, triangulation, member checking, peer review and structural coherence. Transferability was ensured through the purposive selection of the participants, a description of the demographics of the participants, and a dense description of the results with direct quotations from participants. Consistency was ensured through code and re‐code data analysis, a dense description of the research methodology, and peer examination; an audit trail and reflexive notes were used to establish the conformability of the study.

## 3 Findings

### 3.1 Description of the Participants' Demographic Information

The study included 10 participants, both males and females, between the ages of 35 and 40. Most participants were females (7), males (3) and both participants were Tshivenda speakers and resided at Tshisaulu village. Many participants are between the ages of 30 and 35 years (6), followed by those between the ages of 36 and 40 years (4). All participants are biological parents raising children with ID, and many of them had left school after passing Grade 12 (5), followed by those who left school after passing Grade 11 (3), and only one participant left school after passing Grade 8. Only one participant furthered his studies and obtained a Further Education Certificate. Most participants (07) are unemployed, followed by two (2) participants who had full‐time jobs, and one participant is doing a part‐time job Parents pointed out the challenges faced by parents raising children with ID in the selected village in Limpopo Province, South Africa. These themes *(Social challenges faced by parents raising children with intellectual disability, psychological challenges faced by parents raising children with intellectual disability, and Coping strategies employed by parents raising children with intellectual disability)* emerged from data analysis. Sub‐themes that emerged from the *social challenges faced by parents raising children with intellectual disability* include isolation, stigma and lack of understanding and acceptance by society; Sub‐themes that emerged from the *psychological challenges faced by parents raising children with intellectual disability* include stress, depression and anxiety; Sub‐themes that emerged from the *coping strategies employed by parents raising children with intellectual disability* include family support, faith‐based support, professional support and accepting child's condition (see Table 1).

**TABLE 1:** Theme and sub‐themes reflecting the challenges faced by parents raising children with intellectual disability in the selected village in Limpopo Province, South Africa.

Themes	Sub‐themes
Social challenges faced by parents raising children with intellectual disability	1.1 Isolation 1.2 Stigma 1.3 Lack of Understanding and acceptance by society
2 Psychological challenges faced by parents raising children with intellectual disability	2.1 Stress 2.2 Depression 2.3 Anxiety
3 Coping strategies employed by parents raising children with intellectual disability	3.1 Family Support 3.2 Faith‐based Support 3.3 Professional support 3.4 Accepting Child's condition

## 4 Social Changes Faced by Parents Raising Children With ID

### 4.1 Isolation

Parents reported that isolation was one of the social challenges they faced when raising children with ID, the participants reported that their children were isolated from playing with others, this is supported by the following statement.

Most of the time I hide my child inside the house. He likes to go play with other children, but I saw that as soon as he goes out, other children laugh at him. (Participant 1, Female, 31 years).

When he goes outside to play with others, they side‐line him because he attends special school (Participant 2, Female, 31 years).

When he goes to play with other children, they do not want to play with him, they say he is a problem (Participant 9, Female, 35 years).

### 4.2 Stigma

Majority of participants reported that they faced stigma because of their children's condition. They have reported that their child was bullied through name‐calling, and this was supported by the following statement.

Other children she plays with do not understand her, they mock her, and I can see that this does not sit well with her (Participant 4, Male, 35 years).

When my first born goes out to play, he will just come home crying and when you ask him what is going on, he will say that others are bullying him telling him that his brother is mentally disturbed (Participant 1, Female, 31 years).

People here in the village started with talks, laughing at my child, saying I have given birth to a mentally disturbed child, and saying a lot of gossips about my child (Participant 2, Female, 31 years).

People also call her names such as slow learner or dump and I see that this is affecting her (Participant 4, Male, 35 years).

### 4.3 Lack of Understanding and Acceptance by Society

Many parents reported that lack of understanding and acceptance by society is one of the challenges they faced when raising children with ID. The participants indicated that the majority of people in the community do not accept their children, and they always ask questions about the condition of their child; this was supported by the following statement.

People in the community always come and ask me about how I manage to live with my child and whether I don't get tired of her (Participant 5, Male, 39 years).

I feel fed up because I cannot live my life every day explaining my child situation to people (Participant 5, Male, 39 years).

## 5 Psychological Challenges Faced by Parents Raising Children With ID

### 5.1 Stress

Many parents have reported stress as one of the challenges they face when raising children with ID. The participants reported that they stress a lot because they must look after their children all the time and don't have to work and have a boyfriend, and this was supported by the following statement.

This is stressing a lot because I also want someone to spend my time with, when I try to have a boyfriend, it does not work, because I cannot attend my boyfriend and leave my child alone (Participant 3, Female, 40 years).

I have a challenge that is stressing me. I also work at night, so I have a problem because when I hire someone to help here at home, they don't spend a lot of time, they are always leaving the job (Participant 8, Male, 40 years).

This is very stressing me because I am unable to work, I have to look after him every time, and sometimes I feel like my mind is not working well (Participant 9, Female, 35 years).

### 5.2 Depression

The study shows that most participants reported that they faced depression because of their children's condition and lack of support from their children's father. The following statement supports the findings:

I remember when my child was still a few months old. I was admitted to the hospital because I had depression, as I trusted the father of my child, and now, I have a child with him, and he is now turning his back on me (Participant 1, Female, 31 years).

The father of my child used to mock me with my child, saying that at his family they do not give birth to people who are not mentally well, this is when the depression started (Participant 3).

I had a depression especially thinking that I had to start going up and down with doctors, I was unable to cope at first (Participant 10, Female, 34 years).

### 5.3 Anxiety

Anxiety is one of the challenges that most participants reported when raising children with ID. The participants have reported that they worry too much about their child's condition and how people will treat their children. They worry about leaving their child as well as their child's future. This is supported by the statement below:

I had a baby who was very slow in learning, and how to walk. I was very worried and fearing that she would be like him (Participant 6, Female, 38 years).

I always think that if I go back to school to further my studies. I am worried about what will happen to my child, and they will always call me and tell me about my child, and I will not be able to concentrate (Participant 7, Female, 30 years).

I was always afraid and worried that other children will mock him about his condition. This is my biggest fear. (Participant 9, Female, 35 years).

## 6 Coping Strategies Employed by Parents Raising Children With Intellectual Disabilities

### 6.1 Family Support

Many participants reported that family support is one of the coping strategies they are using to cope well with their challenges. This is supported by the following statement:

I get support from my sister because she understands me, because when I call her and ask her to come over, she does not hesitate (Participant 1, Female, 31 years).

I also have support from my family, we support each other very much, even my brother used to fight with the people in the community who bully us (Participant 2, Female, 31 years).

My mother is my pillar, she also shows me the way to raise my child, and she gives me courage to love my child (Participant 3, Female, 40 years).

### 6.2 Faith‐Based Support

Participants have reported that faith‐based support is one of the coping strategies that helped them to cope with the challenges they face. This is supported by the following statement:

I also thank the pastor's wife at church because she is the one who helps me a lot, she gives me counselling and hope (Participant 3, Female, 40 years).

When I talk to the pastor, I sometimes forget about all these challenges I face about my child (Participant 4, Male, 35 years).

I also get a lot of counselling from my pastor, and my brothers support me. They always tell me that I must be strong because a child is a gift from God. (Participant 9, Female, 35 years)

### 6.3 Professional Support

Many participants indicated that professional support is one of the coping strategies they are using to overcome their challenges. This is supported by the responses below:

You know the time I had a depression; I was given counselling by social workers. I was taught to accept, and that god gives us differently, and I am not the first person to have a child with intellectual disability (Participant 1, Female, 31 years).

I have support from teachers at the day care centre because this is where he spends most of his time at. They are with her and be able to check up on her (Participant 5, Male, 39 years).

### 6.4 Accepting Child's Condition

The majority of participants indicated that they are coping with the disability of their children because they have accepted their children's condition, and this is supported by the following responses:

I am coping because I have told myself not to listen to what other people say about my child, especially asking me about the condition my child has (Participant 5, Male, 39 years).

God has blessed me with children. I must accept that my life is now based on taking care of these children alone. (Participant 6, Female, 38 years).

He sometimes even forgets that I have called him. So, I have learned to understand him bit by bit (Participant 10, Female, 34 years).

## 7 Discussion

An emerging body of literature has shown that people with disabilities are facing public stigma in various spheres of life (Trani et al. 2020). Similarly, the current study found that raising a child with ID results in isolation. In most cases, parents hide their children inside the house because other children laugh at them, hurting their feelings. The parents in this study further reported that they face stigma because of their children's condition, and their children are bullied through name‐calling. A study by Pryor and Reeder (2015) (cf. Trani et al. 2020) agrees that friends and family members of stigmatised individuals are faced with stigma by association, resulting in avoiding contact with or hiding the stigmatised individual. The same was true for studies conducted in African countries showing that parents caring for their loved ones with ID are in isolation, with limited support from other family members and authorities (Aldersey et al. 2016; MacKenzi and McConkey 2019). These difficulties increase the burden of care and quality of life for caregivers. In the village where the study has been conducted, most individuals have little awareness of ID, and the parents do not assume the responsibility to educate their children, as they too do not understand the condition, which perpetuates stigma and derogation. In agreement with the current study, Scior (2015) agree that Africa is one of the world regions where pejorative terminology, stigmatising beliefs and discriminatory practices towards people with ID continue to exist. Misconceptions about the causes and abilities of people with ID have been identified as key drivers of stigma in African regions (Rohwerder 2018). There are embedded cultural beliefs that disability is caused by spiritual forces or misdeeds from others, and a perception of people with ID as a burden to family/community resources. As shown in this study, in response to the stigma that children with ID experience, their parents hide themselves from social gatherings due to their behavioural problems. The current study also shows that stigma not only affects the parents and the children with disability but also affects the family as they are being bullied by others in the community due to their family member's condition. In support of this finding, Zechella and Raval (2016) agree that families also experienced difficulties in the community or from neighbours who could not understand or accept the condition of a child with ID. Morset (2016) reasons that the considerable lack of understanding and awareness regarding the causes of disabilities and their resulting characteristics is a key factor in the stigma experienced by people with disabilities in Africa. As such, Jansen‐van Vuuren and Aldersey (2020) suggest that community acceptance and belonging for people with IDD are end‐goals for anti‐stigma interventions. Socio‐cultural studies also argue for the promotion of inclusive practices in education and community settings to challenge stigma and promote positive change (Cui 2023; Jansen‐van Vuuren and Aldersey 2020). In the context of the current study, since most community members lack understanding of ID, educating them on ID to dispel misconceptions will promote positive change and understanding of the condition. The strategy will limit the concerns parents have about being asked uncomfortable questions about their children's conditions and the way they manage to live with ID children.

Due to the special needs of their children, parents reported experiencing stress of staying home with their children and not being able to find employment. The study has revealed that parents of children with ID are more likely to be stay‐at‐home moms than those with non‐disability because they must be home fully looking after their children. In support of this study, Lias (2019) indicates that parents feel stressed to bear the heavier caregiving demand, and the constant dependence of care results in parents spending a lot of time and energy managing their children's daily routine. Contrary to this study, a recent study pointed out that raising a child with ID can generate positive outcomes for a family, including improved family closeness, personal growth, and, importantly, joy (Beighton and Wills 2017). In this study, most mothers were left to care for their children with disability because the biological fathers did not want to be associated with the child. As a result, the mothers were depressed by the demand of caring for the child alone without emotional or financial support. Recent studies also noted breakdown of family relationships, with some mothers reporting the separation from their husbands, which has been linked with excessive care requirements (Aksamit and Wehmeyer 2022; Rodrigues et al. 2019). Scherer et al. (2019) found in their systematic review that parents of children with ID are at a high risk of depression and anxiety. In this study, the parents reported continuous fear and worry about their ID children's condition and future, fear of leaving their children under the care of someone else to further their studies. The parents also fear and worry about their children being mocked or bullied by other children who do not have a disability. Kalgotra (2016) indicated that parents' anxiety is higher on parents raising children with ID because they tend to worry about their children's future. Karisa et al. (2021) reason that some parents prefer to take care of their children on their own to ensure their safety, as they will always think about what will happen if they leave their child with other people. However, the majority of the parents in this study relied on support from their immediate family members. This is supported by the study of Heiman (2016) on parents of children with disability, which reported that most parents tried to cope with stress by taking advice from family members who gave them words of encouragement and assistance in taking care of the child. Additionally, in this study, parents' reliance on religion to overcome the challenges they face when raising children with ID is evident. The parents revealed that talking to the pastors and pastor's wife has helped them to cope well as they provide counselling which gives them hope.

In support of these findings, Kamaruddin and Mamat (2015) indicated that parents are more likely to turn towards religion, engaging in prayer or reassurance, and increased religious activities to reduce stress and challenges. The parents also sought professional support as a strategy to overcome their challenges. The study revealed that professionals such as social workers, teachers and doctors provided the parents with counselling and support, giving them hope and helping them to understand their child better. In support of these findings, Dalmau‐Montala et al. (2017) indicates that collaborations between professional care providers and the families of children with ID would enhance professional support through mutual respect and communication while also enabling these families to acquire appropriate skills and knowledge concerning the care of their child with ID. Parents felt that taking assistance from professionals helped them reduce their challenges and stress over time in managing their children with intellectual disabilities. The parents also reported that to cope with their challenges, they have accepted their children's condition. Most parents have learned to live with their children's condition, and they have adjusted themselves according to the needs of their children. This is supported by the study of Heiman (2016) on parents of children with disabilities, which reported that many parents had an optimistic outlook, a realistic view and acceptance of their child's disability. The current results show that in a rural setting, parenting children with ID requires a collective effort, which will also help combat stigma. The African proverb befits the study results that it takes a village to raise a child. The ecological model plays an important role in dealing with public stigma and promoting inclusion. The entire ecosystem (family, community, government, etc.) plays an important role in promoting inclusiveness for children with ID (Jacobs et al. 2018). The current study strengthens the existing literature that a multi‐layered approach is important for improving the quality of life of caregivers raising children with intellectual disabilities (Modula 2022; Montowska et al. 2025; Halstead et al. 2018). When parents receive adequate support, as reflected in this study's findings, their mental health and overall well‐being improve significantly. This places them in a better emotional and psychological position to effectively care for and support their children, enabling more positive parenting and improved outcomes for both the parent and the child.

The findings have serious implications for mental health professionals to adopt family‐centred approaches to address concerns of caregivers to avoid co‐parenting in isolation without their spouses. Additionally, culturally sensitive approaches to deal with misconceptions of ID and strategies to promote social change and inclusiveness of children with intellectual disabilities are required. In an African context, the Ubuntu approach should be extended to also include children with ID (the approach of showing care and love). The solidarity that rural communities can adopt to show care and love towards the ID children and their families can eradicate the complication of mental illness arising from parenting challenges.

## 8 Conclusion

The current study discovered that raising a child with ID leads to isolation whereby parents and their children are isolated in the community they live in. Parents hide their children in the house, and they also isolate themselves from the public. This study also revealed that children with ID are being stigmatised by others in the community. They are also labelled, and that causes more challenges to the parents. The current study further revealed that raising children with ID leads to psychological challenges such as stress, depression and anxiety. The study further revealed that parents faced a lot of stress because the children require a lot of attention, and they must spend most of their time with their child and not have their own social life, and are unable to work because they must fully take care of their children. Parents are depressed because they are unable to talk about their challenges since society will judge them, and raising their children alone, they must spend most of their time going up and down with the child. Most parents are constantly worried about the future of their children.

The current study also revealed that parents used family support as a strategy to overcome the challenges they have. They relied on faith and putting their trust in their spiritual parents and religion for support and counselling. They also turned to professionals such as social workers, doctors and teachers for counselling to understand their child better. And lastly, the current study found that parents are overcoming their challenges by accepting their children condition and learning to live with them. The findings show that providing awareness to the community about the condition will reduce the negative attitudes and stigmatisation the parents face. It certainly takes a village to raise a child, and the community in which the parents find themselves with their children, if they are supported and accepted, the children will thrive.

## 9 Limitations and Recommendations of the Study

In this study, there were noteworthy methodological limitations, including sample size, which limits the generalisability of the study findings. There was gender biasness, since the study consisted of more females than males. The results might have been impacted by this gender imbalance, especially when the majority of females have reflected biological absence in the children's lives, while there were males who had their partner's support. The study was also limited to 1 day care centre school for children living with disability, perhaps an inclusion of more centres would have yielded further insight. Different forms of social support programmes that would help the parents cope with a child with disability should be put in place in the communities. Studies have also shown that parents have also experienced depression. Therefore, this study recommends that the government invest in mental health screening programmes for parents in their homes to identify those who are at risk, and proper intervention must be implemented. Furthermore, this study has found significant findings that show that parents raising children with ID face social challenges, especially in the community they live in. The study recommends implementing community‐based awareness initiatives on ID. It also proposes that mental health care providers collaborate with community members to develop and deliver information‐sharing programmes on disability, especially in rural areas. This will help reduce bullying, isolation and stigma about children with ID. There is a need for an ongoing parental support programme to empower parents in their journey of raising a child with disability, especially in rural areas where there are limited resources. This may help parents know that they are not alone in their journey. Future studies can explore the same phenomenon using a large sample and across different ethnic groups. Furthermore, researchers can explore the informal and formal support systems available to families, which may also provide valuable insight into how parents navigate caregiving responsibilities, as this was limited in the current study. In addition, comparative studies across different rural settings could help to identify context‐specific barriers and inform the development of more responsive and inclusive support interventions.

## Author Contributions

M.N. was involved in data collection and data analysis, M.C. was involved in article write‐up and V.B. was involved in protocol development, and article write‐up.

## Funding

The authors have nothing to report.

## Conflicts of Interest

The authors declare no conflicts of interest.

## Data Availability

The data that support the findings of this study are available on request from the corresponding author. The data are not publicly available due to privacy or ethical restrictions.

## References

[jar70257-bib-0004] Aksamit, D. , and M. Wehmeyer . 2022. “Mothers of Adults With Profound Intellectual Disabilities Experiences Their Everyday Life? A Qualitative Retrospective Study From Poland.” Journal of Policy & Practice in Intellectual Disabilities 19, no. 3: 257–269.

[jar70257-bib-0127] Aldersey, H. M. , H. R. Turnbull , and A. P. Turnbull . 2016. “Family Support and Self‐ Help in Intellectual and Developmental Disabilities in Kinshasa, Democratic Republic of the Congo.” Journal of Policy and Practice in Intellectual Disabilities 13, no. 1: 23–32. 10.1111/jppi.12143.

[jar70257-bib-0014] Beighton, C. , and J. Wills . 2017. “Are Parents Identifying Positive Aspects to Parenting Their Child With an Intellectual Disability or Are They Just Coping? A Qualitative Exploration.” Journal of Intellectual Disability Research 21, no. 4: 325–345. 10.1177/1744629516656073 PMC5703033 27352854

[jar70257-bib-0025] Cui, J. 2023. “The Impact of Stigma on People With Disabilities: A Systematic Review.” BCP Social Sciences & Humanities 21: 108–113.

[jar70257-bib-0130] Dalmau‐Montala, M. , A. Balcells‐Balcells , C. Giné Giné , et al. 2017. “Cómo Implementar el Modelo Centrado en la Familia en atención Temprana.” Anales de Psicología 33, no. 3: 641. 10.6018/analesps.33.3.263611.

[jar70257-bib-0037] Ghana Statistic Service's . 2014. “Population and Housing Census Summary Report of Final Results: Ghana Statistics Service”.

[jar70257-bib-0039] Global Burden Diseases . 2019. “Diseases and Injuries Collaborators: Global Burden of 369 Diseases and Injuries in 204 Countries and Territories. A Systematic Analysis for Global Burden of Disease Study.” Lancet 20, no. 4: 0140‐6736.

[jar70257-bib-0041] Halstead, E. J. , G. M. Griffith , and R. P. Hastings . 2018. “Social Support, Coping, and Positive Perceptions as Potential Protective Factors for the Well‐Being of Mothers of Children With Intellectual and Developmental Disabilities.” International Journal of Developmental Disabilities 64, no. 4–5: 288–296. 10.1080/20473869.2017.1329192. PMC8115529 34141317

[jar70257-bib-0042] Heiman, T. 2016. “Parents of Children With Disabilities. Resilience, Coping, and Future Expectations.” Journal of Developmental and Physical Disabilities 14, no. 2: 159–171.

[jar70257-bib-0051] Jacobs, P. , K. MacMahon , and E. Quayle . 2018. “Transition From School to Adult Services for Young People With Severe or Profound Intellectual Disability: A Systematic Review Utilizing Framework Synthesis.” Journal of Applied Research in Intellectual Disabilities 31, no. 6: 962–982. 10.1111/jar.12466. 29932264

[jar70257-bib-0052] Jansen‐van Vuuren, J. , and H. M. Aldersey . 2020. “Stigma, Acceptance and Belonging for People With IDD Across Cultures.” Current Developmental Disorders Reports 7, no. 3: 163–172. 10.1007/s40474-020-00206-w. 32837827 PMC7326393

[jar70257-bib-0053] Kalgotra, R. 2016. “Stress and Anxiety Among Parents of the Children With Intellectual Disabilities.” Indian Journal of Health & Wellbeing 7, no. 4: 427–429.

[jar70257-bib-0054] Kamaruddin, K. , and N. Mamat . 2015. “Stress Among the Parents of Children With Learning Disabilities: A Demographical Analysis.” International Journal of Humanities Socials Sciences and Education 20, no. 4: 194–200.

[jar70257-bib-0129] Karisa, A. , J. McKenzie , and T. De Villiers . 2021. “Their Status Will Be Affected by That Child’: How Masculinity Influences Father Involvement in the Education of Learners With Intellectual Disabilities.” Child: Care, Health and Development 47, no. 4: 517–524. 10.1111/cch.12864. 33675047

[jar70257-bib-0055] Katherene, S. 2021. “Pastoral Care and Intellectual Disability: A Person‐Centred Approach, University Press.” Journal of Disability & Religion 26, no. 3: 355–356.

[jar70257-bib-0058] Kromberg, J.,. E. , P. Manga , A. Venter , and E. Rosen . 2008. “Intellectual Disability in the Context of South African Population.” Journal of Policy and Practice in Intellectual Disabilities 29, no. 2: 89–95.

[jar70257-bib-0062] Lias, K. 2019. “Risk and Resilience Among Mothers and Fathers of Primary School Age Children With ASD in Malysia: A Qualitative Constrictive Grounded Theory Approach.” Frontiers in Psychology 9, no. 5: 1–20. 10.3389/fpsyg.2018.02275 PMC6331527 30670992

[jar70257-bib-0065] MacKenzi, J. A. , and R. McConkey . 2019. “Intellectual Disability in South Africa: The Perspectives of Family Cares in South Africa”. 10.1111/jar.12209 26358760

[jar70257-bib-0072] Mazzuchelli, T. , M. R. Sanders , L. Studman , and T. Robert . 2006. “Behavioural Family Intervention for Children With Developmental Disabilities and Behavioural Problems.” Journal of Clinical Child and Adolescent Psychology 35, no. 2: 180–193. 16597214 10.1207/s15374424jccp3502_2

[jar70257-bib-0077] Modula, M. J. 2022. “The Support Needs of Families Raising Children With Intellectual Disability.” African Journal of Disability 11: a952. 10.4102/ajod.v11i0.952. PMC9257718 35812770

[jar70257-bib-0080] Montowska, L. , R. Plinta , and M. M. Gruszczyńska . 2025. “Perceived Life Satisfaction of Parents Raising a Child With Intellectual Disabilities.” Annals of Agricultural and Environmental Medicine 32, no. 4: 552–557. 10.26444/aaem/203276. 41472592

[jar70257-bib-0082] Morset, M. 2016. “Stigma as a Barrier to the Implementation of the Convention on the Rights of Persons With Disabilities in Africa.” African Disability Rights Yearbook 4, no. 6: 2–24.

[jar70257-bib-0088] Nigeria Demographic and Health Survey . 2018. “National Population Commission: Abuja, Nigeria”.

[jar70257-bib-0125] Pryor, J. B. , and G. D. Reeder . 2015. “Stigma: Implications for Helping Behaviour.” In The Oxford Handbook of Prosocial Behaviour, edited by D. A. schroeder and W. G. Graziano , 433–456. Oxford University Press. 10.1093/oxfordhb/9780195399813.013.020.

[jar70257-bib-0093] Rodrigues, S. A. , B. J. B. Fontanella , L. R. S. de Avó , C. M. R. Germano , and D. G. Melo . 2019. “A Qualitative Study About Quality of Life in Brazilian Families With Children Who Have Severe or Profound Intellectual Disability.” Journal of Applied Research in Intellectual Disabilities 32, no. 2: 413–426. 10.1111/jar.12539. 30353627

[jar70257-bib-0128] Rohwerder, B. 2018. “Disability Stigma in Developing Countries (K4D Help Desk Report, 2018).”

[jar70257-bib-0096] Scherer, N. , I. Verhey , and H. Kuper . 2019. “Depression and Anxiety in Parents of Children With Intellectual and Developmental Disabilities: A Systematic Review and Meta‐Analysis.” PLoS One 14, no. 7: e0219888. 10.1371/journal.pone.0219888. 31361768 PMC6667144

[jar70257-bib-0099] Scior, K. 2015. “Towards Understanding Intellectual Disability Stigma: Introduction.” In Intellectual Disability and Stigma Stepping Out From the Margins, edited by K. Scior and S. Werner . Palgrave Macmillan.

[jar70257-bib-0106] Statistics South Africa . 2014. “Census 2011: Profile of Persons With Disabilities in South Africa.” Viewed 21 August 2015.

[jar70257-bib-0112] Trani, J. F. , J. Moodley , P. Anand , L. Graham , and M. T. Thu Maw . 2020. “Stigma of Persons With Disabilities in South Africa: Uncovering Pathways From Discrimination to Depression and Low Self‐Esteem.” Social Science & Medicine 265, no. 1982: 265. 10.1016/j.socscimed.2020.113449. PMC7576188 33183862

[jar70257-bib-0113] UNICEF . 2012. Seen, Counted, Included. Using Data to Shed Light on the Well‐Being of Children With Disabilities. United Nations Children‘s Fund.

[jar70257-bib-0118] WHO , and World Bank . 2011. World Report on Disability. WHO & World Bank. http://www.who.int/disabilities/world_report/2011/report.

[jar70257-bib-0121] World Bank . 2020. Disability Inclusion in Nigeria: A Rapid Assessment. World Bank.

[jar70257-bib-0124] Zechella, A. N. , and V. V. Raval . 2016. “Parents Children With Intellectual Disability and Developmental Disabilities in Asian Indian in the United States.” Journal of Child and Family Studies 2, no. 25: 1295–1309.

